# ‘I think this is where this lovely word “sustainability” comes in’: Fruit and vegetable growers' narratives concerning the regulation of environmental water use for food production

**DOI:** 10.1111/soru.12437

**Published:** 2023-05-02

**Authors:** Chloe Sutcliffe, Jerry Knox, Tim Hess

**Affiliations:** ^1^ School of Water, Energy and Environment Cranfield University Bedford UK; ^2^ Science and Collections Division Royal Horticultural Society, Woking Wisley UK

**Keywords:** fruit and vegetable production, Goffman, irrigation, supply chains, sustainability, water framework directive, water regulation

## Abstract

This article concerns UK commercial fruit and vegetable growers’ narratives regarding the sustainability of water use for food production. In it we explore their perspectives on efforts by regulators to limit agricultural withdrawals of water from the natural environment in line with EU Water Framework Directive objectives, alongside their views on retailer sustainability commitments. Discourse analysis is used to investigate how the growers contested restrictive regulation, constructed their identities, portrayed other supply chain stakeholders, and conveyed their social relations with them. Using Erving Goffman's theory of frontstage and backstage performances, the implications for the growers’ water management decisions and their internalisation of sustainability agendas for water are examined. Whilst the growers gave accounts of purposely misrepresenting their water withdrawal practices and their discourse illustrated significant polarisation between environmental and agricultural interests, their underlying commitment to environmental sustainability was ambivalent, with both anti and pro‐environmental attitudes expressed. The growers also frequently gave critiques of superficial sustainability in fresh produce supply chains. We argue that, given contemporary shifting definitions of agricultural identities, settings in which their construction is negotiated can provide windows of opportunity for conventional growers to engage in genuine pro‐environmental performances that may deepen their assimilation of environmental goals and commitment to sustainable water use.

## INTRODUCTION

### Empirical context: Environmental water quality, agricultural water use and regulation

Surface water quality across Europe is under increasing pressure from high chemical loads. Monitoring shows that safe thresholds are regularly exceeded, posing health risks to aquatic environments and water users. Agriculture is a primary cause of water quality issues due to runoff from farmland and reductions in river flow caused by the withdrawal of water from lakes, rivers and aquifers for irrigation (Knox et al., 2020; Wolfram et al., 2021). In 2000, the European Water Framework Directive (WFD) (European Commission, 2000) was adopted to enhance the sustainable management of environmental water bodies across Europe. It has guided regulatory framework development governing all water withdrawals, including those for agriculture (Moss, 2008). However, challenges with achieving the objectives of the WFD have been reported by many, including strong resistance to its implementation from agricultural sectors (Laurenceau et al., 2020; Linton & Krueger, 2020; Mostert, 2020; Ptak et al., 2020; Tsani et al., 2020; Vito et al., 2020).

Water in rivers, streams and aquifers is referred to as ‘environmental water’ to distinguish it from water taken from the public water supply or harvested rainwater. In England, abstraction (withdrawal) of environmental water is the main reason for up to 15% of rivers (especially globally important chalk streams) failing to meet ‘good ecological status’, and is a major cause of damage to wetlands (Environment Agency, 2018). Irrigation predominantly uses environmental water (DEFRA, 2011) and although it accounts for only 1%–2% of all environmental water abstraction, it is a consumptive use, meaning abstracted water is not returned to the same water body. It occurs in the driest parts of the country and at the driest time of year (Hess et al., 2010). Consequently, the environmental regulator—the Environment Agency—has been seeking to ‘end damaging abstraction’ by making changes to abstraction licences where they have the greatest impact on environmental water resources (DEFRA, 2017). This has involved a process of reviewing and modifying abstraction licences, including making licence renewal contingent on efficient use of water (DEFRA, 2019; OFWAT, 2011).

Irrigation enables growers of high‐value fruit and vegetables (including potatoes) to meet the high‐quality requirements of their customers. Licensing changes can limit the productive potential and the associated value of agricultural land, and hence many growers seek to protect their licensed water rights to support future business production needs (Leathes et al., 2008; Lumbroso et al., 2014). In coming decades, rising irrigation demand, increasing domestic and industrial pressures on limited freshwater resources and the concomitant rising risk of abstraction restrictions will continue to challenge growers’ security of access to environmental water for irrigation (Knox et al., 2018; Salmoral et al., 2019).

In recognition of the significant environmental impacts that agriculture can have, farmers are increasingly required to incorporate support for ecosystem services in their agricultural management activities (Herzon et al., 2018; Ingram et al., 2013). For example, agri‐environment schemes have been employed in the UK as a ‘key policy instrument for the delivery of sustainable management of the countryside’ (Ingram et al., 2013, p. 267). However, in line with a growing body of sociological research emphasising the importance of culture and identity in the context of agricultural decision‐making (Burton et al., 2020), researchers have critiqued the assumption that economical rationality alone drives growers’ environmental behaviours (Walder & Kantelhardt, 2018). Research suggests participation in economically‐oriented schemes does not deeply embed pro‐environmental attitudes amongst farmers, which would be necessary for longer‐term agri‐environmental sustainability gains (Burton & Paragahawewa, 2011). In the context of environmental water quality, research by Thomas et al. (2019) has highlighted farmers’ ambivalence towards assuming responsibility for the health of water bodies on and around farmland. Therefore, enhancing understandings about the extent to which farmers hold pro‐environmental attitudes relating to water use is necessary for designing effective policy and regulation going forward. In this article, we aim to build understanding of the prospects for contemporary regulatory approaches to manage abstraction sustainably in the UK, via a consideration of how the interviewed growers presented their cultural values and attitudes (Burton, 2004; Burton et al., 2020), social and professional identities (Hervé et al., 2020) and relations with other fresh produce supply chain stakeholders (Dowd et al., 2014; Zurek et al., 2020). We demonstrate how the construction of narratives to strategically resist regulation and legitimise the use of environmental water for agricultural production potentially undermines sustainability policy and examine how the politics of sustainability in agriculture intersects with processes of identity expression and construction amongst agricultural producers.

### Article overview and structure

We use discourse analysis to explore the role of identity as a determinant of management decisions concerning water use for irrigation amongst a sample of fruit and vegetable growers in the UK. We aim to yield insights into the growers’ perspectives on the politics of sustainable water governance within food systems and to identify the implications for their decisions regarding water management and collaboration with regulatory authorities. A set of four questions concerning how the growers presented themselves in relation to others in the food system guides our analysis. First, we explore how the growers presented their professional identities, including their beliefs about how farming is perceived by the general public and politicians. We then look at how the growers presented their relationships with the environment and environmental regulators. Following this, we consider the discourse the growers used about actors in the onwards supply chain and issues around food system sustainability. Last, we recount how the growers shared inside stories around their own environmental water use and irrigation management.

We use Goffman's (1959) concepts of backstage and frontstage performances (meaning displayed social behaviours) alongside social identity theory to analyse the growers’ discourse. This reveals the complexity of their identity expression, with traditional articulations of professional agricultural identities destabilised by wider political discord over the role of agriculture in the contemporary pursuit of an environmentally sustainable future. The growers’ narratives illustrate (i) greater allegiance with commercial interests in the food system, (ii) embattled relationships around the use of environmental water for food production and (iii) an implicit acceptance of pervasive impression management (the deliberate manipulation of appearances) in the context of food system sustainability. All this currently leads some growers to strategically misrepresent their use of environmental water to regulators in order to shore themselves up against restrictive legislation. However, several of the interviewed growers also expressed personal environmental values and an openness to altering social in‐group boundaries (by, in Goffman's terms, shifting the regionalisation of their social performances), illustrating the presence of space for the redefinition of their relationships with the environment and environmental regulation.

### Theoretical context: What has identity got to do with sustainable water use in agriculture?

Identity is understood to influence behaviour (Simons, 2021). Increasingly, research has focused on the role of identity as a key determinant of environmental behaviour amongst farmers (van Dijk et al., 2016) and the need to enhance farmers’ environmental identities if agri‐environmental sustainability goals are to be realised (Zemo & Termansen, 2022). In particular, social identity theory highlights the influence of ‘group membership on environmental attitudes and behaviour’ (Fielding & Hornsey, 2016, p. 2), suggesting that identification with one in‐group can lead ‘members to act in more or less pro‐environmental way’ (ibid.). Social identity theory has been widely applied within social psychology studies examining environmental behaviour amongst farmers (Fielding et al., 2008; Lokhorst et al., 2011; van Dijk et al., 2016). It is applicable to understanding the uptake of sustainable practices since intergroup tensions often characterise environmental issues, and alignment with a particular in‐group can significantly affect environmental outcomes (Fielding & Hornsey, 2016).

Discourse analysis is useful for understanding how social identities are articulated interpersonally. Humans communicate to convey ‘what kind of people we are’, and we often use language to position ourselves relative to others by signalling closeness and similarity or distance and difference (De Fina, 2011, p. 263). As such, discourse analysis provides a window into how individuals align themselves with or differentiate themselves from social groups. However, social constructionism highlights that identities are neither stable nor enduring (despite our experience of them as such) but in a process of continual renegotiation that is highly socially contingent (Dell, 2016; Dick, 2005). We express our identities differently depending on social context and who we are speaking to at the time, and therefore identity expression should not be taken at face value.

In this context, Goffman's dramaturgical approach is relevant since it analyses social interactions theatrically, detailing how performers (social actors) carefully manage their props and settings to present an ideal version of themselves to audiences frontstage, yet often relax their attitudes and practices when out of view backstage. Social actors articulate their own identities in relation to the identities of others within their narratives and in relation to the audiences for whom they are performing. An ideal impression in one context will not be ideal in another, and as such, performers adapt their performances according to the norms and expectations of different audiences and contextual frames (Hargreaves, 2016), in a process that we refer to as impression management. The three components of Goffman's micro‐analytical approach: the performer, the audience and the frame (Dell, 2016), map closely onto three key foci for critical discourse analysis (Fairclough, 2010): the identity, the relational and the ideational aspects of discourse (Dick, 2005). Utilising these analytical categorisations connects the minutiae of social interaction with macro‐level discourse, thereby situating personal presentations of the self within larger‐scale societal structures of meaning and political power (Dell, 2016). Goffman's approach complements social identity theory since he states that ‘the team and its members rather than “individuals” should be the natural unit for our consideration’ (Goffman, 1959, p. 149). He suggests paying close attention to how individuals and groups use language and social cues to indicate whether their behaviours are taking place psychologically within a front or backstage region since this marks out others as ‘team’ members (who share the region backstage) or positions them as the ‘audience’. We will argue that shifts in the regionalisation of the performances given by the growers interviewed mark the opening of pathways that can lead to both local and societal political change around issues of food system sustainability.

Goffman's dramaturgical approach is also highly applicable in the morally endorsed political context of environmental sustainability. The sustainability of agricultural production has been noted as a highly malleable concept for which many different interpretive frames exist (Van Gorp & van der Goot, 2012). The moral standards entailed by the concept of sustainability are particularly subject to impression management, especially where commercial interests promote the imperative that brands appeal to consumers with ethical concerns (Cho et al., 2018; Goffman, 1959; Solomon et al., 2013). Following Goffman's assertions, whilst stakeholders in food supply chains may present themselves as committed to sustainable environmental water management, micro‐analysis can examine how their private actions backstage may not follow suit.

## METHODS

This article is based on the qualitative analysis of a set of semi‐structured interviews that we carried out in 2018 with commercial fruit and vegetable growers whose operations relied upon the availability of supplemental irrigation. All the growers had production operations that were predominantly based in the east and the southeast of England, UK regions that are highly exposed to the risk of water shortages due to low rainfall, high rates of evapotranspiration and drought‐sensitive soils. We aimed to speak to growers with large supplemental irrigation requirements who were responsible for production in areas where environmental water shortages and concomitant regulatory risks were likely to place pressures on irrigation management decisions. As such, the growers’ operations were comparatively large (e.g. cultivated land holdings amongst the vegetable growers ranged between 1200 and 10,000 acres). Land use arrangements included estates, family farms, tenancies and contracting. Conventional management (large‐scale mechanised production utilising synthetic fertilisers and pesticides) predominated, although a small minority engaged in organic production on a sub‐portion of their land. Data collection was part of a broader project exploring ways to increase resilience to water‐related risks in the UK fresh fruit and vegetable system.

Potential participants were identified via online searches and through previous contact with the project research team. We invited a total of 47 growers to participate via an email explaining the rationale for the study and completed interviews with 30 participants. Interviews were nearly all conducted at the participants’ farm offices (with the exception of one conducted by telephone) by one of two interviewers following the same semi‐structured interview guide. This ensured that all interviews covered the same basic topics, although differences in the identities of the two researchers may have elicited qualitatively different responses from the research participants. The topics covered included water‐related risks, technical irrigation management, environmental water regulation, sales and the onward supply chain and sustainability and resilience within the food system. Interviews were audio‐recorded and then transcribed and pseudonymised alphabetically, and care was taken to redact information that would facilitate identification of the participants by those outside the research team. Transcripts can be accessed online at Cranfield University's online research data repository (cord.cranfield.ac.uk). Ethical approval for the research was granted by the Cranfield University Research Ethics System (CURES/3651/2017). The qualitative analysis was conducted using NVivo software (Gibbs, 2002).

### The interview as a ‘setting’ for identity construction

Interviews are considered a key site for identity construction since they provide an audience and therefore a ‘way for people to assume their personhood in social reality’ (Dell, 2016, p. 574). In the cultural context of farming, it has been recognised that ‘the research interview is one of identity work’ (Thomas et al., 2019, p. 372), and the transcripts analysed here provide many insights into the identity construction and positionality of the growers. Dick (2005) emphasised the potential for research interviews to provide insights into the performance of ‘frontstage’ (Goffman, 1959) identity work because power dynamics position interviewees as accountable to the interviewer wherein the former's identity is ‘at stake’, inducing efforts to present a ‘creditable’ front (Dick, 2005). In respect of this understanding, in this article, we consider the interviews as a ‘setting’, (or stage or scene) wherein identity work is performed (Goffman, 1959). Goffman's (1959) dramaturgical approach is useful for exploring how the growers communicated issues around sustainability since it promotes a focus on the strategies they used to manage impressions.

## SETTING THE STAGE FOR IRRIGATION—HOW DID THE GROWERS PRESENT THEIR AGRICULTURAL WATER USE IN THE CONTEXT OF THE DRIVE FOR ENVIRONMENTAL AND FOOD SYSTEM SUSTAINABILITY?

A narrative summary follows of the key findings from the grower interviews with respect to the following four questions (which were also set out in the Article Overview and Structure section):

- How did the growers present their professional identities, including the way they believed that they were perceived by others (see first results section)
- How did the growers present their relationships with the environment and environmental regulators? (see second results section)
- How did the growers present the onwards supply chain and food system sustainability? (see third results section)
- How did the growers present the implications of all this for their environmental water use? (see fourth results section)

The results sections are linked to the pseudonymised transcripts by the provision of alphabetical codes in brackets throughout the text.

### Multifaceted agricultural identities: From productivist precision agriculture to environmental stewardship

National food security (especially in terms of availability and affordability of fresh fruit and vegetables to the consumer) formed a touchpoint in many of the discussions, wherein growers highlighted the small but significant contribution of their own production output to national food provisioning at certain times of year (C, D). Several growers also emphasised that there could be a risk of ‘empty shelves’, leading to commercial disaster for supermarkets (A, F) if the regulator was to ‘turn the tap off’ (D). Although most of the potato supply to the UK is homegrown, half of the vegetables and 90% of the fruit is imported (Hess & Sutcliffe, 2018). This high reliance on imports was presented as a risk to national food security: ‘If there's a global food shortage, each country will concentrate on feeding themselves… I think it is very, very concerning from a UK point of view that we are so obsessed with imports’ (K).

Growers also discussed imports from an environmental sustainability perspective, suggesting lower domestic production levels from restricted access to environmental water could result in more imports from overseas (D), with the potentially worse environmental impacts of importing produce from more arid areas highlighted:

You know the water supply was being recharged at half a percent a year, so as soon as they sucked that dry, it was going to be years and years and years before it built up. But they are allowed to do it, and those spuds are on our shelves now and that does my head in. That's so wrong! (A)

Engaging in moral discourse around imports permitted producers to present their claims to environmental water favourably, not only because their production activities enhance food security but also in light of ethical questions around importing food whilst potentially exporting drought to more water‐scarce production locations overseas. Their self‐identification as providers of food security links with ideals around the intensification of production to provision a key public good (the availability of food) that chimes with traditional understandings of what it means to be a ‘good farmer’ (Burton et al., 2020; Cusworth & Dodsworth, 2021).

Yet, despite moral assertions about the provision of food security, all the growers indicated that, in response to pressure from supermarkets, their over‐arching priority was actually the production of high‐quality fruit or vegetables rather than high yields: ‘The major retailers have quality standards, and if you don't meet them, they won't take the produce’ (S). Several growers emphasised that this goal far outweighed the traditional production aim of achieving high yields: ‘I'm interested in growing quality. Quantity doesn't mean anything. If you haven't got quality you are buggered’ (C). There was wide agreement that having sufficient irrigation available was therefore of critical importance to the growers’ ability to grow quality fruit and vegetables: ‘You don't get quality without lots of water’ (A), with one grower admitting ‘water is being wasted to promote quality rather than the mass that you eat when you get it at the end’ (C). Growers also reported instances of significant pre‐farm gate wastage that contradicted moral discourse around food security. One reported ploughing in carrots due to market oversupply making harvesting uneconomic (C). Another discussed the risk of having to throw away fields of edible potatoes that failed quality standards due to scab (N). As such, the growers’ comments revealed aspects of impression management in their self‐presentations from the outset as well as the role of contemporary market pressures in destabilising the identification of agriculture with its traditional primary objective of food provision for the public good.

Growers engaged in discourse that highlighted their hard work and mastery of machinery. Expansion of production areas means farm managers are responsible for larger and more complex irrigation operations, necessitating that they never take their ‘eye off the ball’ (D). In this way, growers emphasised that the role of the farm manager has shifted: ‘I've worked a hell of a lot harder than my father ever did (no disrespect to him)’ (A). All the growers discussed the need to master new irrigation technology, including input from technical soil moisture monitoring equipment, with one grower describing himself as simply another part of the computer network: ‘I'm just basically the server. I take information from whoever inputs it into me and then I just push it to wherever’ (M). All reported efforts to increase irrigation efficiency by investing in new application and monitoring equipment. However, despite these investments, none of the growers indicated any intention to reduce the overall volume of water they would use in their production efforts. Instead, aligning with ideals around economic growth and productivity, they universally indicated that any water savings due to enhanced efficiency would be used to increase production:

Well, as it gets more and more commercialised and our volume goes up, I don't think we are going to use less water, we are going to produce more with less. So it's likely to go up, but we want to be more efficient, we want to produce more kilograms per litre of water used. (V)

Grower narratives thus indicated how agricultural identities were changing over time, highlighting a shift towards greater alignment with illustrations of competence in precision farming, reflecting findings from Burton et al. (2020, p. 60). Emphasis on efficiency, productivity and intensification, however, places this type of identity expression at odds with environmental objectives to conserve water resources (Cusworth & Dodsworth, 2021).

In this context of increasing mechanisation, growers emphasised the risk of losing connection with the land (D) and the danger of simply ‘farming the office’ (C). Many stressed the importance of continuing to walk the fields and ‘feel the soil’ using a spade or their hand to check soil moisture: ‘And then there's the age‐old thing of feel the soil, smell it, squeeze it. Experience. It's a dark art as I'm sure others will have conveyed!’ (J). Such descriptions signalled that authentic lived experience of farming retained significant devotional meaning despite shifts towards lower levels of human presence in the fields. This highlights the risk of a loss of emotional resonance as farming identities increasingly align with highly technical precision agriculture.

Further conveying connectedness to the land, growers presented framings of the farm manager as an environmental steward: ‘We are helping the environment, we are custodians of the countryside!’ (M). In an emotional power play, one grower linked this notion of care for the land with food production to counter the expectation that farmers can provide environmentally sustainable food at very low prices: ‘You are better off to just shut the door, look after the asset, and wait until the f***ing world realises it does need feeding!’ (O). Even those engaged in contracting, whose association with the land they farmed was of limited duration, presented themselves as operating in an environmentally responsible way. The following comment illustrates a recognition of negative portrayals of agricultural land use as damaging and irresponsible and manifests a desire to resist and reshape these perceptions via the articulation of sustainable land management:

The old adage was, you go in, you rape the land, and you go away again. Well, that's not what we are about anymore, you know. We all want to do our bit to preserve the land. If we can improve it, it's a bonus. (P)

Several growers made comments that conveyed their impression that public and political support for farming was dwindling, acknowledging the existence of negative discourse labelling modern agriculture as potentially harmful. One indicated that he believed that the public viewed farmers negatively due to media portrayals of their responsibility for historical food safety crises such as salmonella and BSE: ‘We've not got a great press, have we?’ (H). Two others referred to increasing animosity between farms and neighbouring rural communities on account of the residents’ perceptions of safety risks from increasingly industrial farming activities (L and C): ‘Locals get very concerned about this amount of lorry traffic’ (L). Conventional agricultural production was felt to be at odds with the current political environmental agenda, especially root vegetable crop production that requires significant soil cultivation: ‘In theory, if you follow the ultimate government policy, you would stop growing vegetables in the UK and import them all’ (L). This sentiment was echoed by another grower who suggested the government would rather rely on imports to preserve the UK's natural environment, ‘They could manage us like a glorified fun fair, if you like, or park. There'd be no water to clean up from insecticides, phosphates, slug pellets, all those things…’ (B). These comments convey concern that rural land is being reframed as a place for conservation and leisure, rather than as a productive working environment in line with agricultural management (Burton, 2004), and highlight the anxiety that traditional agriculture and agricultural identities are threatened by environmental narratives.

This section has explored the ways in which environmental conservatism and productivism (which are increasingly concerned with profit rather than yield) sit together uncomfortably within contemporary conventional agricultural identities, leading to increasingly antagonistic discourse about what constitutes ‘good farming’ in the context of environmental sustainability.

### Agricultural identities and ‘The Environment’: Political tensions and contested knowledge

Despite presenting themselves as environmental caretakers, growers constructed ‘The Environment’ as an adversary pitted against agricultural interests. Several referred to past disputes over water resources, highlighting antipathy towards ‘the environmental lobby’ (C). Two expressed animosity towards a leading conservation charity due to historical disputes, ‘We don't talk about [the conservation charity] here. Um… they are very unwelcome’ (F). Another attributed difficulties with licence renewal in his area to a court case over whether abstraction was causing a reduction of sphagnum moss in a local protected habitat. One grower quipped, ‘Ooh, the environment! Ooh, that's the E‐word. You'll have me swearing again!’ (H), exaggerating his performance to humorously acknowledge the existence of a strained relationship between farming and the environment. Grower narratives thus designated environmental actors and the political concept of ‘The Environment’ as players on the opposite team in an emotionally charged arena.

Nevertheless, several of the growers also demonstrated that they intrinsically valued the natural areas on their farms, with the same grower (H) describing his efforts to avoid abstracting water from one of his reservoirs that provided a haven for wildlife:

No, it's because we have created such a lovely place, with an island in the middle and we've got reeds and everything round the outside, and there's fish in it, and we just don't want to bugger it up. (H)

The growers’ representations of the capacities, expertise and scientific knowledge of the environmental regulators they engaged with around water abstraction issues also predominantly revealed tensions and a lack of mutual understanding, ‘They have no idea how an irrigator works. What's involved, how much water is required, all those things’ (A). As such, there was a prevalent belief that regulators poorly understood farmers’ needs. Likewise, the growers mostly attested to having ‘very little’ (S) understanding of regulatory decision‐making about the sustainability of environmental water use at the local (catchment) level.

Whilst there was strong agreement that relations between farmers and the environmental regulator had improved in comparison with the past, some growers expressed dissatisfaction in their dealings with regulatory staff.

The relationship has improved to no end because we can sit down and talk. But sitting down and talking means one thing, getting the action, or the responses that are required, or the explanations as to why you don't get what response you want, is still not an easy job. (B)

Flexible working patterns amongst regulatory staff (including job shares and part‐time working) were highlighted as incompatible with growers’ needs for prompt responses relating to urgent crop water demands. Moreover, these working patterns were in stark contrast to the growers’ own highly pressured schedules. One grower pointed out that whilst good relations could be maintained superficially, they may not stand up well if tested: ‘I'm not knocking them, and in fact, I get on with them very well. But I have to say, how well I'd get on with them if we had a serious issue, I don't know’ (C). Such comments highlighted differences between the operation of frontstage pleasantries in interactions with regulators and more significant backstage concerns around access to water resources.

In response to perceived threats to their abstraction licences, growers constructed arguments that delegitimised regulatory capacity to distribute water effectively and queried the philosophical underpinnings of the current approach. Several participants critiqued the use of Article 1 of the WFD (European Commission, 2000), stipulating the prevention of water body deterioration as the basis for determining abstraction regulation, describing it as ‘completely open to interpretation’, and at risk of being implemented differently elsewhere in Europe (C and G). The so‐called precautionary principle, seen to undergird Article 1 and determine decision‐making on permissible abstraction volumes, was considered problematic: ‘Agriculture is very much pushed aside because the environment has got to come first and they don't do anything because of the… er…yeah, precautionary principle problem’ (M). Such comments reveal the position taken by many growers in response to broader societal processes of polarisation between agriculture and the environment, which one grower acknowledged by commenting: ‘It is understood that “good for the environment” generally means “disadvantage for farmers”’ (G).

Disputing the WFD's tenet of no deterioration in water quality status, several growers constructed the environment as adaptive and resilient to human interference, and therefore, perhaps, not requiring stringent regulation: ‘Well, the thing is “low flow” doesn't happen very often, and I think the equations (or the models) don't take sufficient account of environmental resilience’ (J). Similarly, another grower raised the prospect that shifting ecological boundaries due to climate change would necessarily alter definitions of environmental water requirements anyway. ‘We've got climate change, and therefore the environment will change whether you allow more water in the rivers or not. And they are not recognising that fact’ (B). Growers also complained that regulatory agency definitions of environmental water requirements seemed to be expanding over time, ‘It has been a moveable thing’ (C), with, for example, the recently recognised significance of groundwater for catchment health now increasing regulatory risks for groundwater abstractors who had been relatively untroubled in the past (F and G). Narratives thus emphasized the constructed and therefore politicised nature of scientific knowledge, implying that no claim about the environment should be considered exempt from frontstage manipulation. One interviewee argued that this led growers to represent their water needs strategically in communications with environmental stakeholders, resisting the pressure to give up any of their licensed volumes in order to try to prevent the environmental lobby from attempting to lay claim to more:

This has been the approach. All the farmers’ lobbies have done that…You give them a little bit then they want more. The best thing is to resist giving them anything for as long as possible then you are further back in that chain of them wanting more. It's a psychological thing. (C)

In a related strand of narrative, two of the growers queried the findings of ecological research that had been carried out on their farms and highlighted a mismatch between the slow pace of scientific knowledge development and the fast‐paced needs of farmers operating in the commercial sphere. One criticised the narrow focus of environmentalists: ‘In terms of everybody that gives you problems, they are single‐issue people, so they are not problem solvers’ (O). Another grower told the story of an ecologist he had hired to assess the impact of one of his licences, highlighting the ecologist's refusal to believe his own results when they contradicted his preconceptions about the negative environmental impacts of agricultural abstraction: ‘His argument was, “how can it be better when you are taking water?” Why can't it be better when you are taking water?! Other things come don't they?’ (C). In the same vein, two growers verbally reframed the established scientific consensus around climate change as optional and emotionally driven with one commenting: ‘if you subscribe to climate change…’ (L), and the other attesting to not being a ‘fan’ (C) of global warming. Grower discourse concerning The Environment thus illustrated a significant degree of opposition to the principles underlying environmental water regulation and a prevalent perception of the relationship between agriculture and the environment as highly polarised. Nevertheless, ‘The Environment’ and environmental actors were differentiated from more positive discourse about natural areas and wildlife on and around farmland, illustrating that tensions did not necessarily characterise direct relationships with nature outside of the human dimensions of its governance.

This section has highlighted politically charged tensions between agriculture and the environment, exploring how growers contested scientific claims about environmental sustainability whilst nevertheless admitting to pro‐environmental sensibilities backstage.

### ‘Acting sustainably’: Trust, impression management and inauthenticity in fresh produce supply chains

Despite acute pressure on growers to meet stringent quality standards, sales contracts between customers and growers were described as informal and trust‐based:

You need to build that relationship… you've got to be able to prove over a period of time that you can produce the quality and the quantity on time. And another thing is that whilst we talk about contracts, a lot of them aren't really contracts, they are agreements to supply to a certain level at a certain quality, and the day you don't is the day next year's negotiations start getting harder. (B)

Even the terms of written contracts were easily bypassed ‘you can drive a bus through a contract if you really want to!’ (P). Moreover, contract terms were enforced selectively, taking into consideration a grower's capacity to supply the agreed volumes at the required quality standards over a period of several years rather than on the basis of under‐supply in a single season. ‘They won't look at single years because everybody knows we get peaks and troughs year to year. So, they will try to ride with us’ (O). As such, whilst most contracts stipulated some form of financial penalty for failure to supply, the majority indicated that these penalties were rarely applied. The rare scenario where penalties were likely to be enforced was if a grower was found to be selling ‘out the back door’ (G) (meaning the grower was breaking contract terms in order to benefit from higher prices by selling covertly to a different customer), reflecting the expectation that growers and their regular customers play on the same team and do not dishonour agreements offstage. In addition to discretion over contract enforcement, customers also had the power to flex produce quality specifications according to seasonal conditions, produce availability and their needs at the time. Grower–customer relationships were thus formed with the anticipation that they would endure for years and hinged heavily upon trust being established between parties. Front‐stage ‘rules’ around contracts and produce quality were applied according to backstage considerations on the part of the customer.

Growers indicated they felt most of their customers understood the vagaries of fruit and vegetable production quite well, ‘I call them the grown‐up customers’ (O), which contrasted with grower descriptions of the limited degree to which they felt regulators understood abstraction requirements for irrigation.

Whilst growers depicted a lesser frontstage–backstage divide in their interactions with customers than with regulators, they nevertheless portrayed impression management at other points in the supply chain as rife. This included a fixation on superficial quality standards that did not take account of underlying qualities such as edibility. Several lamented the fact that specifications only concerned characteristics such as size, shape, colour and skin finish: ‘The quality issues that the supermarket and the consumer look at are pure eye candy. They are not looking at internal quality or anything else… Taste, it's only just now coming back into vogue’ (P). Another grower suggested retailers did not care if they supplied produce with poor edibility:

It's… ‘How much bruising you got? How many surface cracks are there? Is the skin nice and shiny? Has it got any scab on it? Yeah, that's fantastic! Oh, eats like shit? Doesn't matter!’ And that is right throughout the board. (H)

Such narratives reflected concerns around the loss of authentic experiential engagement with food, with a yearning to return to seasonal eating expressed by several participants:

What I would like to see is to go back to seasonality. You're too young… but we used to get strawberries… Christ, now I can have strawberries anytime I want. If I want asparagus tonight I can have it, can't I? But it was looking forward to these things! End of April—asparagus is coming! And then the strawberries! And I think we are missing out a lot. (H)

Supermarkets were felt to misrepresent consumer preferences in order to pressure growers to adhere to higher produce specifications: ‘They always say customer demands are higher and higher, but I don't always believe that. I think customers would actually accept a slightly stained onion because they realise an onion isn't perfect’ (E). They were also believed to manipulate consumers: ‘The consumer, I believe, his views are engineered by supermarkets’ (L). In addition to being ‘led’ by supermarkets (O), consumers were thought to be unconcerned about the potential negative impacts of environmental water use for growing fresh produce: ‘Whether many of them think about it more than once in a blue moon, I don't know’ (S). The same was felt to be true of supermarkets, despite their stated concerns about sustainable environmental water use: ‘They want to say all those lovely words, but actually they'll be the ones that will go and buy asparagus from Peru, bang in the middle of the asparagus season in this country!’ (M). In line with this, growers felt that supermarket audits were merely enacted to pay ‘lip service’ (D) to environmental water sustainability, via a ‘tick box’ exercise (E), without a genuine commitment to enhancing sustainable water management amongst growers, illustrated by the fact that no one was ever sent to ‘physically’ check on grower adherence (E). In relation to environmental water use auditing, one grower painted sustainability as a buzzword of limited real‐world utility:

I think this is where this lovely word sustainability comes in. There's this whole topic now, and I find it rather abused and over‐used. But I have to, as part of [the accreditation scheme], show how much water is used per tonne of fruit, and the problem for us… I can do it, but the variable is manifold because it all depends on our season. (S)

The casting of sustainability statements as just ‘lovely’ words reflected the belief that consumers’ espoused preferences for environmental sustainability were mere performances that rarely translated into shopping habits: ‘You know, conscience comes at a price, and there's example after example of when push comes to shove, people's environmental conscience goes at of the window in order to protect their wallet’ (J).

Reflecting this incongruity between the front and backstage of sustainable consumption habits, growers also illustrated an internal incongruence that came into force when they were acting as consumers. Whilst many of the growers indicated holding pro‐environmental attitudes, for example: ‘I'm sorry, I've got a bit of a thing about bottled water and how environmentally unfriendly it is’ (U), and ‘I'm disgusted with the amount of packaging we use. It's all wrong!’ (P), some nevertheless admitted failing to adhere to their frontstage sustainability principles when they went to the shops: ‘But I'm as bad as anybody else, if I feel like having strawberries for tea we'll go and have some strawberries’ (H).

Thus, whilst growers articulated greater alignment with commercial than environmental interests, many also critiqued superficiality and tokenistic ‘sustainability’ within fresh produce supply chains, highlighting that the excessively broad application of the term meant it had come, for some, to signify very little.

### Strategic staging: Managing the frontstage and backstage of environmental water abstraction

Mirroring the theme of impression management within the supply chain, grower narratives frequently revealed a backstage–frontstage divide within their irrigation management decisions and their interactions with regulators around abstraction. They described management decisions that served to limit regulatory efforts to reduce licensed abstraction volumes, gave growers increased access to water for direct abstraction and led to the use of more water than was strictly necessary for producing food.

Narratives emphasised the importance of appearing to use water efficiently: ‘We want to be seen to be using it efficiently. Okay, we want to use it efficiently if we are short of it anyway’ (F). The use of highly visible irrigation application equipment such as rain guns exposed growers to potential criticism (due to higher application rates and susceptibility to wind drift). ‘Clearly, you know, not seeing a lot of your water evaporating or blowing away is quite a nice thing’ (U). Visible aspects of poor irrigation use (such as watering the road) were best avoided, ‘But suddenly we'll have 2 inches of rain when it hasn't even been bloody forecast, and you've just irrigated the field and half of it ends up in the village’ (C). Alluding to the potential for criticism, one grower joked that farmers irrigate at night (a recommended practice for enhancing water efficiency due to lower temperatures and wind speeds), ‘so nobody can see us do it!’ (H).

Describing interactions with regulators around licensing, growers described engaging in various kinds of impression management. One described receiving hydrological advice to determine the best point on a river to position a water meter to limit the risk of abstraction restrictions:

We work with an academic who specialises in rivers and catchments and how they work, and he actually changed all our original applications and told us to apply for our pumping stations somewhere else because he walked the river and said, ‘if you apply there, they'll have a job to refuse you because there's twice as much water there as what there is there’. And I guess that's the sort of advice that you need. (L)

Another grower explained that he avoided aggregating a large number of abstraction licences (which had accumulated as the farm enterprise had expanded over the years) since it made the enterprise's total licensed volume harder for regulators to scrutinise during the review process: ‘My own cynical, personal view is, while it is so complicated it's easier to get them through… They just see this big pile of paper and they just think, “Oh sod it!” and just sign it off!’ (H).

On the basis that abstraction licences were being reissued at volumes matching the highest annual use over a reference time period, growers reported widespread efforts to make it seem as if full licensed volumes were needed, even when this was achieved by irrigating crops that did not really require irrigation: ‘So at the back of my mind, and all the other abstractors, if they've got any sense, will be thinking the same, is in one of those years I've got to pump all of my water’ (O).

Arguing against the loss of headroom (spare capacity) that would be incurred by a reduction in licensed volume, one grower reported being part of a group that had misrepresented the impact of past voluntary abstraction reductions, stating growers in his catchment had previously agreed catchment wide voluntary reductions that didn't ‘really cut in’ (J) due to wider catchment over‐licensing. As such, the growers had presented themselves to the regulator as being willing to forgo a percentage of the water available to them to ease pressure on water resources during a dry period, when this actually had no impact on their ability to irrigate as normal. The participant expressed genuine consternation that if licensed volumes were reduced, such voluntary reduction agreements would no longer be possible.

As such, the growers used their narratives to openly reveal the ways in which they strategically managed frontstage impressions of their agricultural water use with the aim of limiting their potential exposure to the risk of regulatory restrictions.

## DISCUSSION AND CONCLUSION

During their interviews, the bulk of the growers’ performance work focused on constructing narratives to strategically resist regulation and legitimise their right to use water for agricultural production. They claimed the moral high ground by aligning their production activities with arguments around food security, highlighted questionable social‐environmental impacts of overseas fruit and vegetable production and emphasized their own hard work, technical proficiency and the efficiency of their production practices. Yet, aspects of their narratives laid the ground for dramaturgical interpretation (Goffman, 1959). Whilst productivism featured in their frontstage presentations (Burton, 2004), backstage, productivity was secondary to the superficial appearance of produce quality, and statements about the importance of production for food security were weakened by admissions that resources are wasted (both water and produce) in the pursuit of profitability.

They presented the relationship between ‘Agriculture’ and ‘The Environment’ as highly fraught, with many growers opining that agriculture was playing second fiddle to the environment in contemporary politics and had fallen from favour in public opinion. Agricultural perceptions of waning public support have also been reported by researchers working with Canadian farmers (Letourneau & Davidson, 2022), showing that disaccord between food producers and consumers is a common issue within contemporary food systems across national contexts, which signals the increasing ‘development of a critical capacity regarding issues of sustainability amongst the general population’ (Brown, 2016). In the interviews, the growers sought to counter perceived negative portrayals of agriculture, blaming media reporting of food safety crises (Abbots & Coles, 2013); and asserting that their practices are no longer exploitative of the land. The growers commonly aimed to delegitimise environmental regulation, portraying scientific environmental knowledge as subjective and value‐laden, questioning the scientific and philosophical tenets underpinning current reforms and emphasising differences in work ethics and practices between themselves and those working for regulatory or scientific organisations. In order to destabilise the environmental lobby's claims, growers made reference to their own local area knowledge (Carolan, 2006), casting themselves as real‐world problem solvers, in distinction to ‘single‐issue’ people that cause problems for others. By contrast, bias in scientific interpretations was called out (in the case of the ecologist who refused to believe his own findings) and the partial nature of human scientific knowledge was underlined (given that we cannot fully understand the complexity of the natural world). By constructing representations of environmental science that placed the reliability of this knowledge base in doubt, the growers sought to undermine confidence in regulatory conclusions about the necessity of restricting agricultural water use.

By contrast, the growers expressed greater alignment with commercial actors, emphasising trust, mutual understanding and give and take. This contradicts portrayals of conventional agricultural networks as relatively devoid of trust in comparison with sustainable agriculture and alternative food networks (Carolan, 2006; Trivette, 2017). It also illustrates that growers generally positioned customers on the same ‘team’ as themselves (e.g. by stating they ‘ride with us’ (O)). Yet many nevertheless brought the onward supply chain's sustainability failings to the fore in their discussions, in particular highlighting the disingenuous sustainability performances of retailers and consumers. By portraying these behaviours as pervasive in the food system, grower discourse normalised impression management around sustainability within the supply chain. This in turn legitimised their own ‘covert’ backstage efforts to resist environmental water regulation. Meanwhile, relations with regulators were cast as amicable yet shallow and lacking in trust, and the viewpoints of environmentalists were differentiated from agricultural perspectives, demarcating these groups as separate ‘teams’, in Goffman's sense (1959). Growers also applied this distanciation to consumers, who were portrayed as uncaring, hypocritical and easily manipulated by supermarkets (also reflecting findings of consumer ‘othering’ by Letourneau & Davidson, 2022). Direct relationships with consumers, which according to some researchers is a factor that imbues the work of food production with meaning and emotional significance (Baumann et al., 2022), were largely absent in the growers’ descriptions.

Despite a primary emphasis on productivism in their self‐presentations, many of the growers nevertheless did articulate care for the environment in various ways, stressing the importance of a connected and care‐giving relationship with the land and undertaking to protect natural areas on their farms. Later on, when discussing their identities as consumers, some expressed pro‐environmental values and behaviours, worrying about the negative environmental impacts of current production and consumption patterns, denouncing retailers’ over‐emphasis on superficial produce quality attributes and critiquing a perceived lack of customer and consumer concern about some of the fundamentals of food (such as how it is produced and how it tastes).

By attempting to assimilate both productivist and conservationist strands in their identity expression, the growers revealed nuanced and contradictory attitudes towards environmental sustainability. Their comments illustrated the destabilisation of traditional agricultural identities and the difficulty of trying to straddle polarised economic growth‐oriented and environmental ideologies (Letourneau & Davidson, 2022). They acknowledged the risk that the increasing uptake of mechanisation and precision technology means losing traditional connectedness to the land (Giagnocavo et al., 2022) and highlighted the pressure growers face to align with opposing environmental and commercial expectations (Sutcliffe et al., 2021).

On the basis of the growers’ efforts to dismantle environmental knowledge, their stronger identification with the commercial side of the supply chain and their limited allegiances with environmental players, questions may be raised about whether environmental values are currently sufficiently deeply embedded in conventional agricultural identities to ensure self‐driven sustainability gains in future (Burton & Paragahawewa, 2011). Nevertheless, the growers’ allusions to more deeply held values around the importance of authenticity in the food system (their emphasis on the need to care for and connect experientially with the land and the yearning some expressed to prioritise taste over appearance and re‐harmonise consumption patterns with natural seasonal cycles), may open pathways towards greater commitment to environmental protection. Some growers reported experiencing incongruence between their environmental values and their professional or personal behaviours (e.g., ‘…the amount of packaging we use… it's all wrong!’, and ‘…I'm as bad as anybody else, if I feel like having strawberries for tea we'll go and have some strawberries’). Superficial and insincere sustainability performances were raised repeatedly throughout the interviews, demonstrating an acute awareness amongst the growers of the strategic sustainability presentations undertaken by everyone, including themselves. But despite the ubiquity of frontstage behaviours that disguised underlying realities, concerns for genuine environmental commitments were voiced. Individuals experience positive emotions when their values and goals are congruent (Greenebaum, 2012), suggesting opportunities to better align sustainability behaviours with underlying beliefs may be welcomed. Research has highlighted the impact that publicly expressing green credentials can have on environmental behaviour amongst farmers (Howley & Ocean, 2020), implying that narrative assertions about caring for the environment may help reposition underlying commitments backstage. As such, from an identity theory perspective, providing settings wherein conventional growers can publicly articulate and exhibit their pre‐existing environmental values and behaviours may be an important strategy for facilitating moves towards greater sustainability in their professional practices.

Whilst the concept of frontstage/backstage sustainability performances formed a focal point within the grower narratives, the growers also conducted their own impression work in the interviews, delivering performances for the interviewers in the interview ‘setting’. Despite multiple accounts of food system artifice, the growers strove to portray their interview performances as heartfelt via the confessional sharing of ‘inside secrets’ (especially around their strategically staged presentations of environmental water use). Goffman (1959) asserts that sharing inside secrets can serve to demarcate membership of an in‐group. He suggests this ‘lowering of barriers represents a natural phase in the social change which transforms one team into another’ (p. 200). As such, the decision by several growers to recount the strategies they had used to disguise or (mis)represent their environmental water use is significant. Sharing such inside information can be interpreted as an attempt to assign the audience (in this case the interviewers) to new roles as team members. Alongside the growers’ expressions of multivalent environmental attitudes, this willingness to engage in team transformations, joining with stakeholders representing environmental interests (in this case, resilience researchers), may illustrate the availability of ‘space for redefinition’ in their processes of social identity construction (Dick, 2005, p. 1386). Despite the growers’ resistance to the current regulatory approach, the underlying environmental attitudes they expressed reveal counterpoints on which conventional agricultural identities could pivot towards greater environmentalism. Thus, whilst their opposition to environmental water regulation was high, outside of the politicised battle between agriculture and the environment, many of the growers nevertheless did articulate direct environmental care and concern.

Sustainability has been designated ‘an empty signifier’, a term that can be invested with diverse meanings according to politically disparate agendas. By this process, it has been argued that its radical potential for change has been co‐opted by the dominant status quo (Brown, 2016). Nevertheless, this interpretation of sustainability belies the term's capacity to allude to something more meaningful. The growers’ narratives around sustainable water use brought to the fore many diverse incarnations of sustainability, from its empty tokenism within the supply chain to the prioritisation of economic sustainability that drives smaller agricultural businesses out of production, to positivist scientific constructions that validate technocratic governance, to notions of deeper spiritual connection with the natural world. The recurrent theme of misleading superficial appearances illustrated their awareness of social realities as multiply‐layered and raised the problem of which forms of identity and sustainability to place trust in. The many shades of sustainability worn by different actors within different performance regions illustrate that the politics of sustainability are also a politics of identity. Just as there is a difference between what individuals reveal frontstage and backstage, so there is a difference between the signifier and the signified in the realm of sustainability, and one which is widely manipulated by dominant economic forces driving unsustainable practices. The growers commonly drew attention to failures to realise ‘real’ sustainability within contemporary food supply chains, yet their very capacity to make this critique signifies that the potential for successful sustainability does indeed exist, albeit in a space outside of the boundaries of the current system. In the context of agricultural identities, renegotiating the boundaries of social in‐group identification in relation to the environment may offer a strategy for opening up ways into this space.

## CONFLICT OF INTEREST STATEMENT

The authors declare that they have no known competing financial interests or personal relationships that could have influenced the work reported in this article.

## FUNDING INFORMATION

The Global Food Security's ‘Resilience of the UK Food System Programme’, with support from BBSRC, ESRC, NERC and Scottish Government under Grant No. BB/N020499/1

## ETHICS STATEMENT

The interviews on which this article is based were granted ethical approval by Cranfield University's CURES system.

## Data Availability

Redacted texts of the transcribed interviews referred to in this article can be found at https://doi.org/10.17862/cranfield.rd.12033651.
